# Azole-Induced Myositis after Combined Lung-Liver Transplantation

**DOI:** 10.1155/2022/7323755

**Published:** 2022-03-25

**Authors:** Sofie Happaerts, Michiel Wieërs, Ward Vander Mijnsbrugge, Laurent Godinas, Dirk Van Raemdonck, Laurens J. Ceulemans, Robin Vos, Geert M. Verleden

**Affiliations:** ^1^Department of Respiratory Diseases and Lung Transplantation, University Hospital Gasthuisberg, Leuven, Belgium; ^2^Department of Radiology, University Hospital Gasthuisberg, Leuven, Belgium; ^3^Department of Thoracic Surgery and Lung Transplantation, University Hospital Gasthuisberg, Leuven, Belgium

## Abstract

Lung transplant recipients experience a high rate of invasive pulmonary aspergillosis infections, for which voriconazole is the treatment of choice. We report a patient who developed voriconazole-induced myositis that relapsed after one dose of isavuconazole. Our patient was a 38-year-old man who received a single sequential lung transplantation and liver transplantation because of end-stage cystic fibrosis. He presented to our emergency room with acute pain in both forearms at 3 weeks after voriconazole was initiated for invasive pulmonary aspergillosis infection. Levels of voriconazole were normal during the course of therapy. After stopping voriconazole, the symptoms decreased but relapsed after one dose of isavuconazole. Other causes of muscle pain and inflammation were excluded. Magnetic resonance imaging of both arms showed muscle edema in both arms, including involvement of the fascia, consistent with myositis. There were no signs of necrosis. Isavuconazole was discontinued, and the corticosteroid dose was temporarily increased, with rapid resolution of all complaints. Our patient is the fourth reported case of voriconazole-induced myositis, and the first whose symptoms relapsed after initiating isavuconazole.

## 1. Introduction

Lung transplant recipients experience a high rate of invasive pulmonary aspergillosis (IPA) infections. Voriconazole remains the treatment of choice and is generally well tolerated, but a multitude of adverse effects have been described, such as reversible disturbance of vision, skin rashes, photosensitivity, and altered liver function tests [1]. We report here a patient who developed voriconazole-induced myositis.

## 2. Case Presentation

A 38-year-old Caucasian man presented to our emergency room with acute, spontaneous pain in both forearms starting earlier that day, together with a temperature of 38°C. The pain radiated from the elbows to wrists, covering the entire forearms, and was continuously present. He noted a decrease in grip strength, mainly because the movement increased his pain. The pain was alleviated by paracetamol. No other signs or symptoms were present.

### 2.1. History of Past Illness

The patient had a history of cystic fibrosis (CF), homozygotic mutation delta-F508. He had CF-related liver cirrhosis, distal intestinal obstruction syndrome, and diabetes mellitus. He received a combined single sequential lung transplantation and liver transplantation in 2015 at the age of 33 years, which is 4.5 years before the current presentation at the emergency room. The initial immunosuppressive regimen after transplantation consisted of tacrolimus, mycophenolate mofetil, and methylprednisolone. Antifungal prophylaxis was provided with inhaled liposomal amphotericin B 50 mg twice weekly for 3 months after his transplantation. Cytomegalovirus status of the donor was positive, and that of the recipient was negative. Posttransplantation problems consisted of Epstein–Barr virus-associated lymphoproliferative disorder in 2016 and multiple respiratory infections, including IPA in 2016. At that time, he had been successfully treated with voriconazole for 3 months, without any notable side effects.

### 2.2. History of Present Illness

Five days before the emergency room visit, the patient had been discharged from our hospital after a 2-week intravenous antibiotic treatment with a combination of ceftazidime, ceftazidime-avibactam, and tobramycin for a respiratory infection of *Pseudomonas aeruginosa* and methicillin-susceptible *Staphylococcus aureus*. There was a concomitant decline in forced expiratory volume in 1 second. At the same time, he was diagnosed with IPA based on new bilateral ground glass opacities on chest computed tomography (CT) and a bronchoalveolar lavage fluid galactomannan index of 4.0 (optimal optical density index). Treatment with voriconazole was initiated at 21 days before the patient's emergency room visit.  Table 1 shows the discharge medications from his recent hospitalization. Immunosuppressive therapy at that moment consisted of tacrolimus 1 mg daily and methylprednisolone 4 mg daily.

### 2.3. Physical Examination

On clinical examination, the patient had a temperature of 39.1°C. Other vital signs were within normal limits. His conscious state was normal. Manipulation of the cervical spine provoked no pain. Inspection of his arms did not show cutaneous lesions, and there was no visible redness or swelling. Pain could be elicited with minimal touch of the arms. Strength of his upper limbs was slightly diminished on both sides, with a score of 4.5/5 on the Medical Research Council Scale for muscle strength. There was no loss of skin sensitization. Articulation of the joints was normal, as were tendon reflexes. Lung auscultation was normal, and there were no signs of respiratory distress. There were no clinical signs of liver failure nor portal hypertension.

### 2.4. Laboratory Examinations

Upper airway polymerase chain reaction swab test for severe acute respiratory syndrome coronavirus 2 (commonly known as SARS-CoV-2) was negative. Four sets of blood cultures were drawn, all of which were negative. Laboratory results (Table 2) showed mild normochromic anemia, mild thrombopenia, normal white blood cell count, and elevated C-reactive protein (i.e., CRP) of 41.9 mg/L (reference range: <5.0 mg/L). Kidney function was normal, with an estimated glomerular filtration rate of 75 mL/min/1.73^2^. There was no increase in creatinine kinase (i.e., CK).

Tacrolimus trough level was subtherapeutic on admission 2.5 mcg/L, but quickly normalized to therapeutic target range of 5-6 mcg/L after increasing his maintenance dose.

Voriconazole trough level 2 days after presentation was subtherapeutic at <0.2 mg/L (reference range: 2.0-5.5 mg/L). We note that voriconazole trough level 6 days before presentation was within therapeutic range with 2.7 mg/L.

### 2.5. Differential Diagnosis

Our primary differential diagnosis was muscle pain due to voriconazole, because it was the most recently initiated therapy, and pain disappeared after discontinuation. Diabetic muscle infarction was the second differential diagnosis in this patient with CF-related diabetes mellitus. An infectious cause and autoimmune myositis were considered less likely.

### 2.6. Clinical Course

The patient was admitted to the lung transplantation ward, voriconazole was discontinued, and piperacillin-tazobactam was empirically started because of fever and inflammation. Low-dose chest CT scan demonstrated a significant reduction of ground glass opacities compared to the CT scan performed 6 weeks earlier.

Analgesics were given systematically, and the patient recovered quickly. His temperature normalized, and the pain almost completely disappeared. CRP further increased to 181.5 mg/L (reference range: <5.0 mg/L) on day 2 of hospitalization and fell steeply to 71.3 mg/L on day 3. On day 3 of hospitalization, the patient was started on a loading dose of isavuconazole at 200 mg three times a day for treatment of IPA, as he had only received 3 weeks of voriconazole therapy. We chose isavuconazole as alternative for voriconazole because we experience a high tolerability in our lung transplant patients and because of the possibility of oral administration. However, immediately after the first 200 mg dose, the patient reported reappearance of his pain; thus, isavuconazole was immediately discontinued. The next day (day 4 of hospitalization), his temperature rose to 38°C. CRP increased gradually from 33.8 mg/L on day 5 to 179.1 mg/L on day 10 of hospitalization.

### 2.7. Further Diagnostic Work-Up

**Figure 1:**
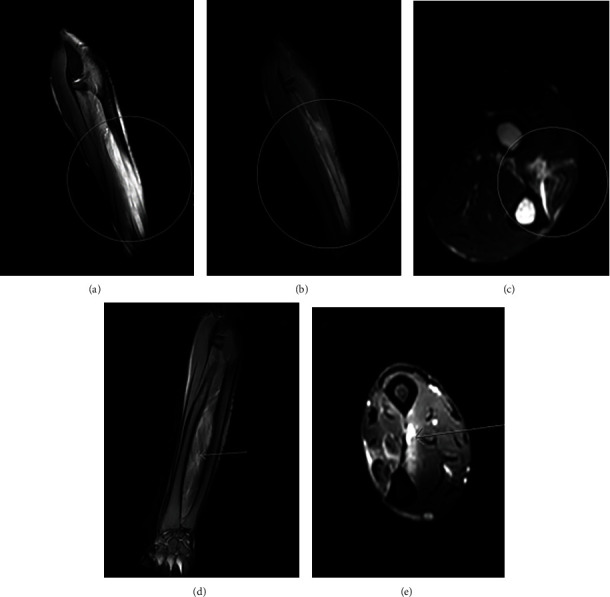
Magnetic resonance imaging of the arms. (a)–(c) Heterogenous T2-hyperintensitivity of the extensor carpi ulnaris muscle of the left arm, with surrounding edema of the fascia and subcutis.(d, e) Heterogenous T2-hyperintensitivity of the deep flexor and extensor muscles, superficial extensor muscles, and extensor carpi radialis brevis muscle. Edema of the fascia was also observed.

During his entire stay, CK levels remained normal, and a selected blood panel of antinuclear antibodies specific for dermatomyositis remained negative. Electromyography of the upper limbs showed no signs of plexopathy. Magnetic resonance imaging (MRI) of the cervical spine did not demonstrate any signs of spinal cord compression or lesion. MRI showed muscle edema in both arms, including edema of the surrounding fascia (Figure 1). These findings did not seem indicative of diabetic muscle infarction considering the absence of necrosis.

### 2.8. Final Diagnosis

The patient's clinical history and MRI scans consistent with myositis were sufficient evidence for the diagnosis of drug-induced myositis.

### 2.9. Treatment

Because of the relapsing drug-induced myositis, we increased methylprednisolone from 4 mg daily to 32 mg daily and tapered the dose over several weeks. We decided to delay further therapy for IPA, as his respiratory complaints, pulmonary function tests, and CT scan were already improving after 3 weeks of therapy with voriconazole. Empirically initiated antibiotics were discontinued after the diagnosis of myositis.

### 2.10. Outcome and Follow-Up

Pain, fever, and inflammation decreased, and the patient was discharged from the hospital 2 days later on day 13. During follow-up in the next several months, the patient had two new episodes of respiratory infection with fever and bronchiolitis-like changes in the lower lobes on chest CT. The first episode was due to *P. aeruginosa* and the second to SARS-CoV-2. In both episodes, there was no evidence of IPA.

## 3. Discussion

Since 2002, voriconazole has been the treatment of choice for IPA. It is a second-generation azole antifungal agent that inhibits the synthesis of ergosterol, the most abundant sterol in fungal cell membranes, by inhibiting the fungal cytochrome P450-dependent enzyme lanosterol 14*α*-demethylase. It is generally well tolerated, but multiple side effects have been described. Most reported adverse events are reversible disturbance of vision (30% of patients), skin rash, photosensitivity, altered liver function tests, headache, nausea, vomiting, diarrhea, and visual hallucinations [1]. Less frequently reported are an increase in cutaneous malignancies (especially in patients on immunosuppressive therapy), cardiac arrhythmias by QT prolongation or electrolyte disturbances, peripheral neuropathy, periostitis (most commonly in ulna and ribs), alopecia, nail changes [2], and myositis. Voriconazole-induced myositis has only been described in three case reports to date [3–5], and one of those cases was also a double lung transplant recipient with a history of CF [4], overview in Table 3.

Drug-induced myopathies are a diagnosis of exclusion. They often present with acute or subacute myopathic symptoms, such as myalgias, muscle weakness, or swelling after exposure to a certain drug. CK can be elevated, and myoglobinuria may occur in more severe cases. Statins, human immunodeficiency virus antiviral therapies (interferon, clevudine), antimalarials, leflunomide, glucocorticoids, and tumor necrosis factor-*α* inhibitors are frequently correlated with myositis [6].

The following criteria to establish the diagnosis of drug-induced myopathies were published in 1991: lack of preexistent muscular symptoms, a free period between the beginning of treatment and the appearance of symptoms, no other cause of myopathy, and complete or incomplete resolution after cessation of the causative drug. Rechallenge therapy is not advisable because of the risk of serious relapse [7].

In our patient, those criteria were all met. As a differential diagnosis, we considered diabetic muscle infarction, a rare microvascular complication in diabetic patients that presents similarly [8]. MRI of our patient's arms was less consistent with diabetic muscle infarction because it showed no signs of necrosis. An infectious cause seemed less likely because both arms were symmetrically affected. The possibility of an autoimmune myositis, such as dermatomyositis, was considered less probable on the one hand because of his treatment with immunosuppressants and on the other hand because a selected panel of antinuclear antibodies for dermatomyositis in the blood was negative.

The gold standard for diagnosing drug-induced myopathy is muscle biopsy, which was not performed in our patient because we concluded that the clinical history and MRI images were sufficient evidence of this disease. We believe that isavuconazole caused a relapse of symptoms. Indeed, the patient experienced very acute reappearance of symptoms and inflammation after administration of a single 200 mg dose. Myositis induced by isavuconazole has not been described in case reports; although, the United States Food and Drug Administration reports that myositis may occur in less than 5% of treated patients [9]. It is a recently approved broad-spectrum azole that has shown noninferiority to voriconazole for invasive aspergillosis therapy in terms of response and survival, and it is considered to have a better safety and tolerability profile [10].

## 4. Conclusion

Physicians who regularly evaluate lung transplant recipients often prescribe voriconazole for probable or definite IPA. More recently, isavuconazole has also become available for the treatment of IPA. Our patient experienced voriconazole-induced myositis, which is a rare adverse event, but should be considered if a patient presents with new muscular symptoms after treatment initiation. Changing the treatment from voriconazole to isavuconazole should be done with caution, as we believe it caused a relapse of the myositis in our patient.

## Figures and Tables

**Table 1:** Discharge medications from the recent hospitalization.

Drug	Dose	Frequency
Alendronate (oral)	70 mg	Weekly
Amitriptyline (oral)	25 mg	Daily
Azithromycin (oral)	250 mg	3×/week
Calcium carbonate (per os)	2.5 g	Daily
Chlorhexidin (mouth wash)		Daily
Colistineb (inhalation)	2 million units	BID
Creon lipase + amylase + protease (oral)	2100 mg	TID
Insulin glargine (subcutaneous)	13 IU	Daily
Insulin aspart (subcutaneous)	3-5-4 IU	TID
Magnesium (oral)	450 mg	Daily
Methylprednisolone (oral)	4 mg	Daily
Metoprololtartrate (oral)	50 mg	BID
Pantoprazole (oral)	40 mg	Daily
Pentamidin (inhalation)	300 mg	Monthly
Sorbitol (oral)	13.8 g	Daily
Tacrolimus (oral)	1 mg	Daily
Ursodeoxycholic acid (oral)	250 mg	TID
Vitamin D colecalciferol (oral)	25000E	Weekly
Vitamin ADEK (oral)	A 2000 IU–D 2000 IU–E 150 IU–K 1 mg	Daily
Voriconazole (oral)	300 mg	BID

**Table 2:** Laboratory results in the emergency room.

Test	Result	Reference range
Hemoglobin	12.1 g/dL	14-18 g/dL
White blood cell count	5584/*μ*L	4000–10,000/*μ*L
Neutrophils	3800/*μ*L	2500-7800/mL
Eosinophils	0 *μ*L	≤400/*μ*L
Basophils	0 *μ*L	≤100/*μ*L
Lymphocytes	1500/*μ*L	1200-3600/*μ*L
Monocytes	500/*μ*L	200-800/*μ*L
Platelets	81,000/*μ*L	150,000-450,000/*μ*L
Sodium (Na)	134 mmol/L	136-145 mmol/L
Potassium (K)	5.74 mmol/L	3.45-4.45 mmol/L
Chloride	94 mmol/L	98-107 mmol/L
Bicarbonate	25.7 mmol/L	22-19 mmol/L
Urea	34 mg/dL	≤49 mg/dL
Creatinine	1.22 mg/dL	0.67-1.17 mg/dL
Estimated glomerular filtration rate	75 mL/min/1.73m^2^	
Albumin	40.4 g/L	35-52 g/L
Total bilirubin	0.66 mg/dL	≤1.18 mg/dL
Aspartate aminotransferase	28 U/L	≤37 U/L
Alanine aminotransferase	19 U/L	≤41 U/L
Gamma-glutamyl transferase	47 U/L	≤60 U/L
Alkaline phosphatase	103 U/L	40-130 U/L
Creatine kinase	24 U/L	≤190 U/L
C*-*reactive protein	41.9 mg/L	≤5 mg/L

**Table 3:** Overview of previously reported cases.

Reference	Gender, age	Toxic agent	SOT	Symptoms	Diagnosis	Management
Shanmugam et al. [3]	F, 34	Voriconazole	Kidney	Generalized weakness	CK ↑, MRI	Discontinuation of voriconazole
Soliman et al. [4]	F, 26	Voriconazole	Lung	Pain left leg	MRI, biopsy	Discontinuation of voriconazole, dose elevation prednisone
Wang and Su [5]	M, 78	Voriconazole	None	Diffuse myalgia, weakness	CK ↑	Discontinuation of voriconazole

## Data Availability

No data were used to support this study.

## Abbreviations

BID: Bis in die
CF: Cystic fibrosis
CK: Creatine kinase
CRP: C-reactive protein
CT: Computed tomography
DIOS: Distal intestinal obstruction syndrome
EMG: Electromyography
FEV_1_: Forced expiratory volume in 1 second
FDA: Food and Drugs Administration
HIV: Human immunodeficiency virus
IPA: Invasive pulmonary aspergillosis
MRI: Magnetic resonance imaging
PCR: Polymerase chain reaction
SARS-CoV-2: Severe acute respiratory syndrome coronavirus 2
TID: Tris in die.
